# Plasma Hepatitis C Virus Viral Load Among Hepatitis C Virus Mono-Infected and HCV/HIV Co-Infected Individuals in Yunnan Province,China

**DOI:** 10.5812/hepatmon.6160

**Published:** 2012-07-30

**Authors:** Xing Liu, Na He, Zhuohua Fu, Song Duan, Meiyang Gao, Zuo Feng Zhang

**Affiliations:** 1 Department of Epidemiology, School of Public Health, Fudan University, The Key Laboratory of Public Health Safety of Ministry of Education, Shanghai, China; 2 Department of Epidemiology, School of Public Health, University of California, Los Angeles, USA; 3 Dehong Prefecture Center for Disease Control and Prevention, Yunnan Province, China

**Keywords:** HIV, Coinfection, Antiretroviral therapy, Highly Active, Viral load

## Abstract

**Background:**

Hepatitis C virus (HCV)/human immunodeficiency virus (HIV) co-infection has become a serious public health problem especially in high risk groups such as injection drug users in China. However, the impact of HIV infection and antiretroviral therapy (ART) on HCV viral load which is usually regarded as a predictor of liver disease progress had not been well studied in this country.

**Objectives:**

To explore correlations of HIV co-infection and ART with plasma HCV load among HCV-infected patients in an ethnic minority area in Yunnan Province, China.

**Patients and Methods:**

HCV/HIV co-infected patients and HCV mono-infected controls were examined and compared for plasma HCV RNA and related risk factors.

**Results:**

A total of 145 HCV/HIV co-infected patients and 25 HCV mono-infected controls were studied. The majority of the participants were male, belonged to an ethnic minority and were younger than 45 years old. HCV viral suppression rate with undetectable plasma HCV viral load was 28.3% in the HCV/HIV co-infected patients, 36% among HCV mono-infected controls and 29.4% overall. ART-prescribed HCV/HIV co-infected patients had significantly higher HCV viral loads (IQR: (3.80-6.44)*log_10_ copies ml-1) than those naïve to ART (IQR: (undetectable-6.41)*log_10_ copies ml-1) and HCV mono-infected patients (IQR: (undetectable-5.44)*log_10_ copies ml-1). Men, from the Dai minority and those with more than six years education, were also shown to have a higher plasma HCV viral load,according to multiple logistic regression analysis.

**Conclusions:**

ART potentially increases the plasma HCV viral load among HCV/HIV coinfected patients in an ethnic minority area in China. Future large scale prospective cohort studies are needed to address the controversy associated between HIV co-infection and the natural history of HCV.

## 1. Background

Patients chronically infected with the hepatitis C virus (HCV) are at high risk of developing liver cirrhosis and end-stage liver disease (ESLD) [1]. This risk could be increased even more by a human immunodeficiency virus (HIV) co-infection, which is very likely to occur due to the shared transmission routes between HIV and HCV [1][2]. On the other hand, HCV co-infection could also accelerate the HIV disease progression among HCV/HIV co-infected patients [3]. Since the HCV RNA level is an important predictor of ESLD among HCV patients [4], it is important to examine the impact of HIV infection as well as HIV targeted highly active antiretroviral therapy (HAART), or simply antiretroviral therapy (ART) on HCV RNA levels among HCV/HIV co-infected patients. However, the existing literature presents controversial results on the association of HIV infections, particularly HIV viral loads with HCV RNA levels, among ART-naïve patients. Some studies have indicated a positive association between HIV co-infection and increased serum HCV RNA levels and liver damage [5], but others have not [6]. Similarly, available studies have observed heterogeneous associations that occur after initiation of ART. The HCV/HIV co-infected patients were more likely to have detectable HCV RNA in peripheral-blood mononuclear cells (PBMCs) [6], or increased plasma HCV titer or RNA levels [7][8][9], or an initial increase in HCV RNA levels followed by a gradual decrease to lower levels than those at pretreatment [10], or no significant changes at all [11]. The reasons for such heterogeneous observations are unclear, but may possibly relate to differences in these studies’ populations, sample sizes and geographical regions. This further underlines the importance and need for such studies in different populations and geographical regions.

In China, the prevalence of HCV infections is relatively low (approximately 3.2%) in the general population [12], however, it is very high among injection drug users (IDU, 11.4% - 90.8%) [13][14] and former commercial plasma donors (FCPD, 9.6% - 72.8%) [15], who have been seriously affected by HIV [16]. So far, there have been no studies specifically designed to examine the association between HIV co-infection and HCV RNA levels or viral replication in China. Moreover, as China has entered the era of ART through the nationwide implementation of a free ART program after 2003 among HIV/AIDS patients, it is time to examine the impact of ART on HCV RNA levels or viral replication as well as liver disease or ESLD among the large number of HCV/HIV co-infected patients in this country.

## 2. Objectives

Therefore, as the first step, we specifically designed and conducted a cross-sectional survey among a group of HCV/HIV co-infected patients and a group of HCV mono-infected controls in an ethnic minority area in the Yunnan Province, southwest China, to examine the correlations between HIV infection and ART with plasma HCV RNA levels among HCV-infected patients.

## 3. Materials and Methods

### 3.1. Study Site

This study was conducted in Longchuan County, one of the five counties in Dehong Dai and Jingpo Autonomous Prefecture in the west of the Yunnan Province near the “Golden Triangle”, where the first HIV outbreak in China was identified in 1989 [17]. Half of the 136 000 permanent residents in Longchuan County are ethnic minorities, with Dai and Jingpo being the two largest minority groups. By the end of 2009, 3 063 HIV/AIDS cases had been reported and approximately half of these patients were infected through injecting drugs, whereas the remainder were infected through heterosexual transmission.

### 3.2. Study Population

Study participants were either infected with both HCV and HIV (i.e., HCV/HIV co-infected) or infected with HCV alone (i.e., HCV mono-infected). The HCV/HIV co-infected participants came from an existing cohort of HIV discordant couples established in this study site since July, 2009 (unpublished study). All of the 265 HIV positive spouses of the cohort were tested for anti-HCV immunoglobulin G (IgG), CD4+ T-cell counts and plasma HIV viral load at the time they enrolled into the cohort. Among the participants, 145 (54.7%) tested positive for anti-HCV IgG and made up the HCV/HIV co-infected group of the present study. The HCV mono-infected controls came from a recently reported community-based cross-sectional survey conducted at the same site in 2009 [18]. All of the 591 adult study participants in this study were screened for anti-HCV IgG. In the group, 25 (4.2%) tested positive and they made up the HCV mono-infected group of the present study. In sum, a total of 170 HCV-infected individuals were included in the present study. Participants venous blood specimens were collected by professional nurses using disposable sterile needles and tubes, the specimens were stored immediately in a cold box and transported to a laboratory within four hours. The blood was centrifuged and the plasma was stored in 500 µL aliquots at -70° C for later use. This study was approved by the Institutional Review Board (IRB) of Fudan University, Shanghai, China.

### 3.3. Blood Testing

3.3.1. Anti-HCV IgG Testing

Anti-HCV IgG was blindly assayed without knowledge of the personal identity of the study subjects, using an enzyme-linked immunosorbent assay (ELISA) technique (Wantai Biomedical Co. Ltd, Beijing, China), according to the manufacturer’s protocol.

3.3.2. HCV RNA Quantification

Plasma HCV viral RNA was extracted and quantified by a real-time polymerase chain reaction (PCR) technique using commercially available kits for the quantification of HCV RNA (PCR-Fluorescent Probing, PG Biotech Ltd., Shenzhen, China). The limit of detection was 500 copies ml-1 and the linear range of HCV RNA quantification was from 1.0 × 103 to 5.0 × 107 copies ml-1.

### 3.4. Other Tests

Quantitative detection of plasma HIV viral RNA was by a NucliSENS EasyQ HIV-1 Assay (bioMérieux, Boxtel, the Netherlands). The linear range of the HIV-1 RNA quantification was from 50 to 3 × 106 IU ml-1. CD4+ T-cell counts were assessed by FACSCount (Becton, Dickinson and Co.,San Jose, CA, USA).

### 3.5. Statistics Analysis

Data were analyzed using SPSS 11.5 for Windows (SPSS Inc., Chicago). The proportion of patients having an undetectable HCV RNA level (i.e., < 500 copies ml-1 in this study) was tabulated by; socio-demographic characteristics, drug use, HIV infection status and receipt of ART. Associations between categorical variables were evaluated by a Pearson Chi-squared test. Univariate logistic regression analyses were performed first, followed by multivariate logistic regression analysis to explore relationships between the listed variables and HCV RNA levels (detectable or undetectable). Odds ratios (OR) with 95% confidence intervals (95% CI) were estimated. In addition, HCV RNA levels were log_10_ transformed to approximate a normal distribution. Median concentrations of HCV RNA were compared with multiple levels of explanatory variables using nonparametric Kruskal-Wallis tests. A significance level of 0.05 was used for all tests.

## 4. Results

### 4.1. Sociodemographic Characteristics 

All study participants who tested positive for anti-HCV IgG were defined as HCV-infected. They had an average age of 37.3 ± 7.4 years old, ranging from 19 to 56 years. Most participants were male (91.8%); 17.1% were Han, the major ethnicity in China, whereas 54.7% were Jingpo minority and 18.2% were Dai minority, which are the two major ethnic minority groups in the study area. The majority (77.1%) of the study participants were illicit drug users. About 82.1% of the HCV/HIV co-infected participants acknowledged illicit drug use, significantly higher than the HCV mono-infected controls (48%). The two groups were also significantly different in gender and ethnicity (Table 1).

**Table 1:** Sociodemographic Characteristics, Drug Use and HIV Disease Status Among Participants of Hepatitis C Virus Viral Load and HIV Co-infection Study in Yunnan, China (n = 170), 2009, 2011

****	**HCV aMono-infected, No. (%) (n = 25)**	**HCV/HIVa Co-infected,** **No. (%) (n = 145)**	**Total, ** **No. (%) (n = 170)**	**χ²**	***P* value**
**Gender**				49.611	< 0.001
Male	14 (56.0)	142 (97.9)	156 (91.8)		
Female	11 (44.0)	3 (2.1)	14 (8.2)		
**Age, y**				2.424	0.298
19-35	10 (40.0)	56 (38.6)	66 (38.8)		
36-45	9 (36.0)	70 (48.3)	79 (46.5)		
46-64	6 (24.0)	19 (13.1)	25 (14.7)		
**Ethnicity**				14.655	0.002
Jingpo	22 (88.0)	71 (49.0)	93 (54.7)		
Dai	0	31 (21.4)	31 (18.2)		
Han	3 (12.0)	26 (17.9)	29 (17.1)		
Other	0	17 (11.7)	17 (10.0)		
**Education, y**				2.165	0.339
0 (Illiterate)	7 (28.0)	23 (15.9)	30 (17.6)		
1-6	13 (52.0)	89 (61.4)	102 (60.0)		
> 6	5 (20.0)	33 (22.8)	38 (22.4)		
**Ever used drugs**				14.000	0.001
Yes	12 (48.0)	119 (82.1)	131 (77.1)		
No	13 (52.0)	26 (17.9)	39 (22.9)		
**CD4+ T-cell counts (cells µl-1)**					
≤ 200	--	23 (15.9)	23 (15.9)		
201-350	--	35 (24.1)	35 (24.1)		
351-500	--	40 (27.6)	40 (27.6)		
> 500	--	47 (32.4)	47 (32.4)		
Median (IQR)		389 (280.5-553)	389 (280.5-553)		
**HIV viral load (IU ml-1)**					
< 50 (undetectable)		45 (31.0)	45 (31.0)		
50-10^5^	--	79 (54.5)	79 (54.5)		
> 10^5^	--	21 (14.5)	21 (14.5)		
Median (IQR)	--	2 800 (< 50-37 000)	2 800 (< 50-37 000)		

^a^ Abbreviations: HCV, hepatitis C virus; HIV, human immunodeficiency virus

### 4.2. Disease Status of HIV/AIDS

The HCV/HIV co-infected participants had an average CD4 + T-cell count of 445 cells µl-1, ranging from 29 to 1 516 cells µl-1 (median: 389 cells µl-1) and a median HIV viral load of 2 800 IU ml-1, ranging from undetectable (< 50 IU ml-1) to 1.6 × 106 IU ml-1 (Table 1), nearly a half (46.9%, or 68/145) of the HCV/HIV co-infected participants were receiving ART. They were more likely to have lower CD4 + cell counts (< 500 cells µl-1) than those without ART (77.9% vs. 58.4%, χ² = 6.268, P = 0.012). However, they were more likely to have undetectable HIV viral loads (i.e., < 50 IU ml-1) in their plasma than those without ART (60.3% vs. 5.2%, χ² = 51.221, P < 0.001).

### 4.3. HCV Viral Load among Participants

Plasma HCV viral load varied from undetectable (< 500 copies ml-1) to 4.0 ｘ 107 copies ml-1 among the study participants. After log_10_ transformation of HCV RNA levels, significant differences were observed between the men (median = 5.17) and women (median = 3.09) (P = 0.003) and in the levels of people with different HIV infection and ART status (P = 0.010) (Table 2). Furthermore, among the 25 HCV mono-infected controls, 14 men showed a significantly higher mean rank of HCV viral load (16.14) than the 11 women (9.00) (P = 0.014). Among the 156 male participants, 65 who were HIV-infected and receiving ART showed a higher mean log_10_ transformed level rank (88.62) of HCV RNA levels than those 14 HIV-uninfected (71.96) and 77 HIV-infected men without ART (71.14), with a P value of 0.057.

**Table 2:** Hepatitis C Virus Viral Load Among Study Participants of Hepatitis C Virus Viral Load and HIV Co-infection Study in Yunnan, China (n = 170), 2009, 2011

	**Participants, No.**	**HCV^a^ Viral Load (log_10_)**		**Pvalue**^b^
		**Median**	**IQR**	
**Gender**				0.003
Male	156	5.17	undetectable - 6.36	
Female	14	3.09	undetectable - 4.26	
**Age, y**				0.169
19-35	66	4.04	undetectable - 5.90	
36-45	79	5.37	2.92 - 6.30	
46-64	25	4.89	1.56 - 6.45	
**Ethnicity**				0.754
Han	29	5.32	undetectable - 6.42	
Jingpo	93	4.80	undetectable - 6.35	
Dai	31	5.14	4.00 - 6.07	
Other	17	2.92	undetectable - 6.01	
**Education, y**				0.654
0(illiterate)	30	4.88	undetectable - 5.68	
1-6	102	4.88	undetectable - 6.44	
>6	38	5.37	2.18 - 6.01	
**HIV^a^/ART^a^status**				
HIV negatives	25	3.99	undetectable - 5.44	
HIV+/ART-	77	4.70	undetectable - 6.41	
HIV+/ART+	68	5.46	3.80 - 6.44	
**CD4+ T-cell counts (cells µl-1)**				0.054
≤ 200	23	5.00	3.74 - 5.92	
201-500	75	5.42	3.10 - 6.70	
≥501	47	4.89	undetectable - 6.05	
HIV negatives	25	3.99	undetectable - 5.44	
**HIV Viral Load (IU ml-1)**				0.084
HIV negatives	25	3.99	undetectable - 5.44	
<50	45	5.42	3.86 - 6.30	
50 - 105	79	4.81	undetectable - 6.52	
>105	21	5.41	undetectable - 6.07	

^a^ Abbreviations: ART, antiretroviral therapy; HCV, hepatitis C virus; HIV, human immunodeficiency virus

^b^ P values were obtained from a Kruskal-Wallis test.

### 4.4. HCV Viral Suppression

HCV viral suppression with undetectable plasma HCV RNA was achieved among 29.4% (50/170) of the study participants (Table 3), 28.3% (41/145) of the HCV/HIV co-infected participants and 36% (9/25) of the HCV mono-infected controls (χ² = 0.613, P = 0.434). Among the HCV/HIV co-infected participants, the HCV viral suppression rate was significantly lower among those receiving ART (17.6%, 12/68) than those naïve to ART (37.7%, 29/77) (χ² = 7.133, P = 0.008). Such a negative correlation between ART and HCV viral suppression remained significant even after being adjusted for potential confounding variables by a multivariate logistic regression model. ART-naïve HCV/HIV co-infected participants were more likely to experience HCV viral suppression than ART-prescribed patients (OR = 3.10, 95% CI: 1.34-7.16), whereas participants of the Dai ethnicity (OR = 0.18, 95% CI: 0.04 – 0.72) or those with more than 6 years education (OR = 0.25, 95% CI: 0.07 - 0.96) were less likely to achieve HCV viral suppression compared with the Han ethnicity or illiterate participants (Table 3). Women were more likely to achieve HCV viral suppression than men (OR = 4.25, 95% CI: 0.99 - 18.29), at a marginal significance level of 0.052 (Table 3).

**Table 3:** Proportions and Correlates of Hepatitis C Virus Viral Suppression With Undetectable Plasma Hepatitis C Virus Viral Load and HIV Co-infection Study in Yunnan, China (n = 170), 2009, 2011

****	**HCV ^a^ Viral Suppression, Proportion (%)**	**OR (95% CI b)**	**P value**	**OR a (95% CI a)**	***P***^****b****^***value***
**Gender**					
Male	27.6 (43/156)	1.00		1.00	
Female	50.0 (7/14)	2.63 (0.87-7.93)	0.087	4.25 (0.99-18.29)	0.052
**Age, y**					
19-35	37.9 (25/66)	1.00		1.00	
36-45	24.1 (19/79)	0.52 (0.25-1.06)	0.073	0.50 (0.23-1.09)	0.082
46-64	24.0 (6/25)	0.52 (0.18-1.47)	0.217	0.43 (0.13-1.46)	0.175
**Ethnicity**					
Han	37.9 (11/29)	1.00		1.00	
Jingpo	28.0 (26/93)	0.64 (0.26-1.53)	0.310	0.41 (0.15-1.12)	0.081
Dai	19.4 (6/31)	0.39 (0.12-1.26)	0.116	0.18 (0.04-0.72)	0.016
Others	41.2 (7/17)	1.15 (0.34-3.89)	0.828	0.70 (0.18-2.80)	0.615
**Education, y**					
0(illiterate)	33.3 (10/30)	1.00		1.00	
1-6	30.4 (31/102)	0.87 (0.37-2.08)	0.760	0.49 (0.17-1.43)	0.194
> 6	23.7 (9/38)	0.62 (0.21-1.80)	0.380	0.25 (0.07-0.96)	0.043
**HIV infection and ART^a^**					
HIV-	36.0 (9/25)	2.63 (0.94-7.33)	0.066	1.28 (0.34-4.87)	0.720
HIV+/ART-	37.7 (29/77)	2.82 (1.30-6.12)	0.009	3.10 (1.34-7.16)	0.008
HIV+/ART+	17.6 (12/68)	1.00		1.00	

^a^ Abbreviations: OR, odds ratio; CI, confidence interval; HCV, hepatitis C virus; ART, antivetro viral therapy

^b^ 95% CIs and P values were obtained from multiple logistic regression analyses with adjustment for potential confounding effects of other variables listed in the table.

## 5. Discussion

The present pilot study adds to our knowledge about whether HIV infection and ART may have an impact on plasma HCV viral loads or HCV viral replication in a setting with multiple ethnicities in China. Although HIV infection alone seems to have no significant impact on HCV viral replication, ART had a positive impact on HCV viral replication or a negative impact on HCV viral suppression among HCV/HIV co-infected patients. These findings are consistent with several prospective studies which reported increasing HCV viral loads after the initiation of ART at 16, 48 and 96 weeks [7][8][9]. Although the detailed mechanism for the increase of HCV viral load with ART remains unclear, it has been attributed to immune restoration after HAART, which leads to an increased CD4 + T cell level and enhanced immune response resulting in the destruction of infected hepatocytes and an increased HCV viral load [7][8][9]. Since HIV-infected patients had to meet the criteria of having a CD4 + T cell count lower than 200 cells µl-1 for ART initiation; ART-prescribed HIV-infected patients might be more severely immune compromised with weakened viral clearance ability. It was not evident that plasma HCV viral load or RNA levels correlated with CD4+ T cell counts or plasma HIV viral load in this study, although the latter two factors did act as markers and determinants of immune competence in HIV-infected patients. This observation is consistent with several previously published studies [19][20][21], there are also a few studies reporting that HCV viral clearance becomes more difficult among people with lower CD4 + T cell counts (< 200 cells µl-1) [22][23] and the HCV viral load increases with declining CD4 + T cell counts [24]. No significant correlation between HCV viral load and HIV viral load was identified in this study, which was very likely due to the different status of ART prescription among the study participants and the dual effects of ART on HIV viral load and host immune restoration. Some studies have found that HCV viral loads increased with increasing HIV viral loads [3][25], without taking the effects of ART into account. Women were more likely to have low plasma HCV viral loads and to have undetectable HCV RNA levels than men in the study. A similar gender difference in HCV viral load or viral suppression was also observed in a long term follow-up cohort study [23], but this was not found in several other studies [26][27]. Future prospective cohort studies with larger sample sizes and more balanced gender ratios are needed to clarify such controversy.

The present study was conducted in a remote rural area with multiple ethnic minorities in southwest China, and it was found that among all of the ethnic groups, the Dai minority had the lowest rate of HCV viral suppression. This suggests that host genetic factors might play important roles in HCV viral suppression or viral clearance. Previous studies have consistently found that black people are more likely to be HCV-viremic [23][28][29]. In fact, the overall HCV viral suppression rate (29.4%) among the present study participants is higher than that of black people, but comparable to that of non-black people in other studies [23][29].

Given the imperfect specificity of the anti-HCV ELISA Kit, we cannot rule out the possibility of false positives in the study. Therefore, it is possible that the false-positive reactions in the anti-HCV antibody test might have increased the overall proportion of HCV viral suppression among the study participants. However, such misclassifications, if any, in anti-HCV antibody status were most likely to be non-differential across the various comparison groups including groups with different HIV/ART status. Therefore, the odds ratios (ORs) comparing the proportion of HCV viral suppression between the different groups were most likely biased towards the null, i.e., the ORs or the associations would have been even larger and more significant if there had been no such misclassifications or false positives. This study has some limitations. First, because of the nature of cross-sectional studies, we were not able to estimate the duration of HCV infection in this present study. The literature shows that over a long duration (more than 20 years) with an HCV infection, that this correlates with HCV viral loads [27] and increases the risk of liver cirrhosis [30]. In addition, we were not able to examine the dynamic changes of HCV viral loads or RNA levels over the disease progression of HIV infection and with longer time periods after ART. Therefore, our ability to make causal inferences was further limited. Second, the relatively small sample size, especially the small number of women and HIV negative participants in this study, made it difficult to measure associations precisely. Future well designed prospective cohort studies with large samples, will enhance our ability to test these hypotheses by detailing influences which result from the natural history of HIV and HCV infections.
